# Association between carcinoembryonic antigen levels and the applied value of ^18^F-fluorodeoxyglucose positron emission tomography/computed tomography in post-operative recurrent and metastatic colorectal cancer

**DOI:** 10.3892/ol.2014.2523

**Published:** 2014-09-11

**Authors:** WENZHE BU, RAN WEI, JINPENG LI, LIJUN WANG, CONGCONG SHI, JINLONG SONG, SHUANGSHUANG MA, HUA CHEN, NING CONG

**Affiliations:** 1 Department of Surgical Oncology (Interventional Therapy), Shandong Cancer Hospital and Institute, Shandong Academy of Medical Sciences, Jinan, Shandong 250117, P.R. China; 2 Department of Computed Tomography, Shandong Cancer Hospital and Institute, Shandong Academy of Medical Sciences, Jinan, Shandong 250117, P.R. China; 3 Department of Computed Tomography, Shandong Medical Imaging Research Institute, Jinan, Shandong 250000, P.R. China; 4 Six Ward of Shandong Mental Health Center, Jinan, Shandong 250014, P.R. China

**Keywords:** colorectal neoplasm, positron emission tomography, carcinoembryonic antigen

## Abstract

Positron emission tomography (PET) using ^18^F-fluorodeoxyglucose has been widely used for analyzing cellular metabolism. The present study aimed to evaluate the association between the diagnostic value of PET/computed tomography (CT) in patients with post-operative recurrent and metastatic colorectal cancer (CRC), and the different levels of carcinoembryonic antigen (CEA). A total of 105 suspected recurrent and metastatic CRC patients (67 males and 38 females; mean age, 48.5 years) were included in this retrospective study. All the patients underwent PET/CT examination. The differences in the PET/CT diagnostic values of CEA-positive and -negative patients with recurrent CRC following surgery were retrospectively analyzed and compared. Among the 105 CRC patients, 87 exhibited recurrence and metastasis, as confirmed by histopathological diagnosis or clinical follow-up data. By contrast, the PET/CT examination results revealed that 85 cases were true positives (a false positive foci was diagnosed in one of the patients), 18 were true negatives and 2 were false negatives. Correspondingly, the sensitivity and degree of accuracy were 97.7 and 97.1%, respectively. The detection rates of PET/CT for the recurrence and metastases were 85.3% in the CEA-positive group and 75.7% in the CEA-negative group. No significant differences were observed between the two groups. Overall, CEA levels do not help improve the detection rate of PET/CT in the recurrence and metastasis of CRC. PET/CT imaging has a high sensitivity and degree of accuracy in detecting recurrence and metastasis following CRC surgery. Therefore, this method is ideal for monitoring relapsed and metastatic foci of post-operative colon cancer cases.

## Introduction

Colorectal cancer (CRC), which is the second most common cancer in each gender globally, has relatively high local and distant recurrence rates (1). Although radical surgery and post-operative chemotherapy are currently the main treatment for patients with CRC, recurrence following an apparently curative resection remains common, with reported relapse rates of up to 40% (2). Therefore, accurate early detection of recurrence and metastasis is critical to improving the survival rate of CRC patients. Serum carcinoembryonic antigen (CEA) measurement is the most widely accepted method for determining recurrent CRC (3). Measurements of serum CEA and periodic abdominal ultrasound and computed tomography (CT) are useful for detecting recurrent HCC in the early stages. However, rising CEA levels often occur even when conventional imaging studies and clinical examinations are normal. Accordingly, a more sensitive means for localizing tumor foci would aid in the management of such patients.

^18^F-fluorodeoxyglucose (FDG) positron emission tomography (PET)/CT is a functional imaging tool that can provide anatomical and functional information, and has been used for the staging and restaging of several cancers (4). ^18^F-FDG PET/CT has also been used in patients with CRC for staging and restaging. It has been shown in several studies that ^18^F-FDG PET/CT is a sensitive imaging tool in the detection of CRC recurrence in patients with elevated CEA levels (5–8).

The present study aimed to evaluate the association between the diagnostic value of PET/CT in patients with post-operative recurrent and metastatic CRC, and the different levels of CEA.

## Materials and methods

### Patients

Between August 2010 and May 2013, a total of 105 patients (67 males and 38 females) with suspected recurrence of CRC, as observed histologically, by elevated CEA levels or by conventional imaging, were studied using FDG-PET. The mean age was 48 years (33±67 years). All the patients were confirmed with colon cancer or CRC by pathology, consisting of 83 cases of adenocarcinoma, 5 of mucinous adenocarcinoma, 9 of signet-ring cell carcinoma and 8 of carcinoid tumors. PET/CT was performed in all patients at 2–42 months post-resection. Baseline and follow-up CEA levels were available in 105 patients for comparison. The CEA levels were measured within the time of the FDG PET/CT study. According to a study by Tan *et al* (9), these patients were grouped into two categories depending on the CEA level. The CEA level was determined using an electrochemiluminescence immunoassay (Siemens Healthcare Diagnostics, Inc., New York, NY, USA). Values of <5 ng/ml were considered normal. In total, 68 patients with a CEA level of ≥5 ng/ml were included in the CEA-positive group, while the remaining 37 patients were included in the CEA-negative group. Written informed consent was obtained from all patients. This study was approved by the ethics committee of Shandong Cancer Hospital and Institute (Jinan, China).

### ^18^F-FDG PET scans

All patients fasted for 4–6 h prior to PET scan, which was performed using a Discovery LS PET/CT scanner (GE Healthcare, Buckinghamshire, UK). The imaging agent ^18^F-FDG, with a radiochemical purity of >95%, was produced by using a circular accelerator and synthesized automatically by the automated synthesis modules of GE Healthcare. Serum glucose levels were monitored immediately prior to the injection of the ^18^F-FDG; the blood glucose level was <7 mmol/l in all cases. Intravenous ^18^F-FDG was administered at ~5.5 MBq/kg of body weight, and following a 45 to 60-min uptake period, the PET/CT scan was performed. In the PET/CT system, CT acquisition was performed on spiral dual-slice CT, with a slice thickness of 4 mm and a pitch of 1. The image was acquired using a 512×512-pixel matrix and a pixel size of 1 mm. Subsequent to CT, two dimensional (2D) PET acquisition was performed for 2–3 min per bed position. PET data were acquired using a 128×128-pixel matrix, with a slice thickness of 1.5 mm. CT-based attenuation correction of the emission images was used. PET images were reconstructed by iterative method ordered subset expectation maximization (2 iterations and 8 subsets). Following CT acquisition, the table was moved toward the field of view of the PET, and PET acquisition of the same axial range was begun with the patient in the same position on the table. Following completion of PET acquisition, the reconstructed attenuation-corrected PET images, CT images and fused images of matching pairs of PET and CT images were available for review in axial, coronal and sagittal planes, as well as in maximum intensity projections, 2D cine mode.

### Data analysis

All ^18^F-FDG PET scans were visually assessed by two experienced nuclear medicine physicians familiar with the clinical information. On image analysis, the PET/CT findings were interpreted as positive if the focal area of FDG uptake in the lesions was greater than that of the surrounding tissue, except for the physiological uptake of the body (such as the gastrointestinal tract and bladder) on the same plane of the CT images. Any questionable lesions that were detected by PET/CT, but that had no change in location and/or morphology, were considered to be positive if the disease had higher FDG uptake during the delayed scanning. PET findings were interpreted as negative if the FDG uptake was equal to or less than that of the surrounding tissue. In cases of disagreement, a final decision was made by consensus. Maximum standardized uptake values were calculated for suspected lesions. The gold standard was therefore determined either on the basis of histology or on a 6-month follow-up [significant tumor progression according to the Response Evaluation Criteria In Solid Tumors (10,11)]

### Statistical analysis

SPSS version 19.0 (SPSS, Inc., Chicago, IL, USA) was used for the statistical analysis. According to the different levels of CEA, the χ^2^ test was carried out for comparison of the detection value of ^18^F-FDG PET/CT in the patients with CRC recurrence and metastasis. P<0.05 was considered to indicate a statistically significant difference.

## Results

Of the 105 CRC patients who underwent surgery, 87 cases were confirmed with local recurrence or metastasis by histopathology or 6-month clinical follow-up. In these 87 cases, there were 28 cases of liver metastasis, 25 of lung metastases, 30 of local recurrence and metastasis (intra-abdominal organs and tissues, excluding the liver, pelvis and abdominal wall) and 12 of metastasis at other sites (such as the bones, neck, mediastinal lymph nodes and adrenal gland). Cases with multiple metastases were present in this count.

### PET/CT diagnosis of recurrence and metastasis in CRC patients

**Figure 1:**
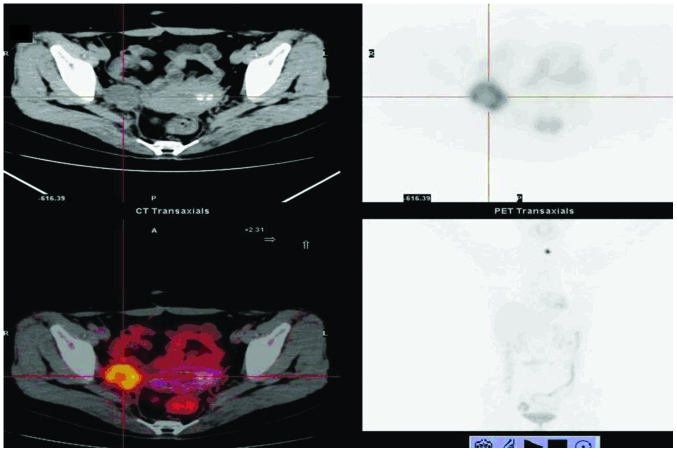
Positron emission tomography/computed tomography (PET/CT) images revealed high-uptake of ^18^F-FDG in the right pelvic lymph node.

**Figure 2:**
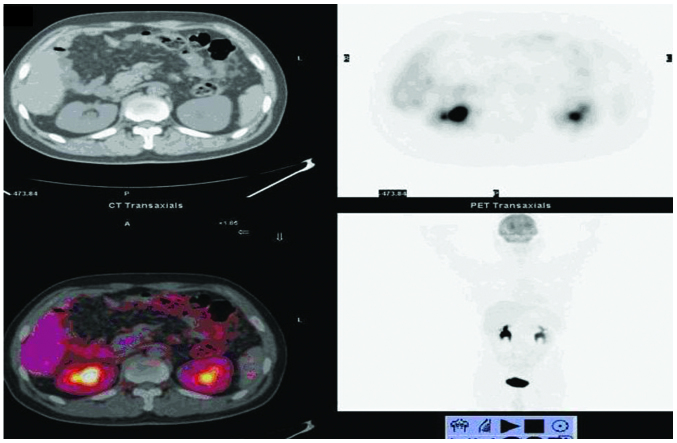
Positron emission tomography/computed tomography (PET/CT) images revealed low uptake of ^8^F-FDG in the retroperitoneal lymph nodes.

A total of 85 patients were diagnosed as true-positives by PET/CT, including one case diagnosed as anastomotic recurrence and right pelvic lymph node metastasis. In this case, anastomosis was proven by inflammatory changes upon biopsy, the high-uptake in the right pelvic lymph node was proven to be a true-positive lesion and the left lobe of the thyroid was found to contain malignant lesions during PET/CT examination; the diagnosis of papillary thyroid carcinoma was confirmed by biopsy (Fig. 1). A total of 18 true-negative cases and 2 false-negative cases were found by PET/CT, including one ^18^F-FDG uptake-negative case, which exhibited severe symptoms of intestinal obstruction and underwent a laparotomy, which confirmed peritoneal metastasis (Fig. 2); another case with retroperitoneal lymph node metastasis was diagnosed as false-negative, as the lymph nodes were too small and due to low uptake. However, this patient was found to exhibit retroperitoneal lymph node metastasis during color Doppler ultrasound examination and lung metastases subsequent to the 6-month clinical follow-up. The sensitivity, accuracy and negative predictive values of monitoring the recurrence and metastasis of patients with CRC by PET/CT were 97.7% (85/87), 97.1% (102/105) and 90.0% (18/20), respectively.

### Detection rate of CEA-positive and -negative groups compared by PET/CT

Of the 105 patients, 87 were confirmed with recurrence and metastasis by histopathology or 6-month clinical follow-up. In the 68 cases of the CEA-positive group, recurrence and metastasis was correctly detected by PET/CT in 58 cases, with a detection rate of 85.3% (58/68). In the 37 cases of the CEA-negative group, 28 cases were correctly detected, with a detection rate of 75.7% (28/37). There was no significant difference (P=0.221) between the PET/CT detection rates for recurrence and metastasis between the two groups of patients.

### Diagnostic value of CEA levels in CRC patients for recurrence and metastasis

In the 68 CEA-positive patients, 59 cases were eventually diagnosed with recurrence and metastasis. In the 37 CEA-negative patients, 28 cases were eventually diagnosed with recurrence and metastasis. The sensitivity of the CEA levels for monitoring the recurrence and metastasis of the patients with CRC was 67.8% (59/87).

## Discussion

CRC is one of the most common cancer entities worldwide. Surgical resection is the optimal treatment for CRC, which is a highly curable disease if detected in its early stages, while recurrence following apparently curative resection remains common, with reported relapse rates of up to 40% (12). Suspected recurrence is present in the first two years of follow-up in ~60% of patients who undergo first curative surgery for CRC (13). Early and accurate detection of such recurrences is not only key to the minimization of subsequent metastatic spread and to the planning of radical surgery, but also to improving the survival rate and outcome of patients to a certain extent.

Serum CEA is a tumor cell adhesion molecule and a useful tumor marker for relapse detection, prognosis estimation and therapy monitoring in CRC patients. Elevated serum CEA levels are detected in two-thirds of patients with CRC. It is possible that tumor recurrence and metastasis may be found prior to the changes in tumor morphology through monitoring the level of CEA. On average, conventional methods localize disease relapse 3–9 months after the elevation of the CEA levels has been documented (14). However, CEA levels are also increased in smokers, inflammatory bowel disease, pancreatitis, liver disease and in patients with epithelial tumors at other sites, but a normal CEA level cannot rule out tumor recurrence and metastasis. The tumor location, distribution, size and other factors affect the CEA level. A prospective study by Moertel *et al* (15) demonstrated only a 25% post-operative recurrence rate in CRC patients with elevated CEA levels. In this study, 24 patients developed recurrence and metastasis in the CEA-negative group, while 4 patients did not develop recurrence and metastasis in the CEA-positive group. Conventional imaging modalities, including CT, magnetic resonance and echography, have limited sensitivity and specificity for the detection of recurrent CRC (16). In general, structural or anatomical imaging modalities encounter difficulties in identifying tumors in the distorted anatomy following surgery and radiotherapy (17,18). The distinction between scar and viable tumor tissue is particularly problematic in previously treated abdominal regions. However, conventional imaging modalities also have certain limitations in monitoring lymph node metastasis, as they cannot distinguish whether enlarged lymph nodes are caused by metastasis, nor analyze certain smaller nodes and lymph node metastases (19,20).

^18^F-FDG PET/CT is a hybrid imaging modality that can provide anatomical and functional information, and has been used for the staging and restaging of several cancers (21). In recent years, it has been used increasingly to identify recurrent disease. ^18^F-FDG PET/CT can not only display the morphology, density and anatomical changes in the lesion, but can also provide chemical information and metabolic functions at the molecular level (22). ^18^F-FDG is the most commonly used imaging agent in PET/CT imaging, and its distribution in the body reflects the level of glucose metabolism *in vivo (*23). FDG preferentially accumulates in malignant tumors and metastatic lesions due to the increased rate of glucose consumption, secondary to the increased rate of glycolysis and cell membrane glucose transporters.

A previous study has shown that PET/CT is extremely accurate for the detection of local and/or distant recurrent disease in CRC patients, with high specificity and sensitivity (24). In this study, the specificity and sensitivity of monitoring the recurrence and metastasis of patients with CRC using PET/CT was higher than that using the CEA level. Certain studies have shown higher diagnostic performances of FDG-PET/CT in comparison with other conventional imaging modalities in the detection of CRC recurrence, particularly in the case of locoregional recurrence and lymph nodes metastases (25). Schmidt *et al* (26) reported that the diagnostic accuracy of FDG-PET/CT was higher than that of whole-body (WB)-MRI in the follow-up of patients suffering from CRC. The overall diagnostic accuracy for PET-CT was recorded as 91% (sensitivity, 86%; specificity, 96%) and that for WB-MRI was recorded as 83% (sensitivity, 72%; specificity, 93%), respectively. Simó *et al* (27) studies assessed the effect of PET/CT detection of recurrent disease on the management of patients with CRC and showed that FDG-PET had a significant impact on the management of patients with suspected recurrence.

In conclusion, PET/CT has a higher diagnostic value for monitoring the recurrence and metastasis of patients with CRC. In the CRC patients with normal or elevated CEA levels, PET/CT showed high specificity and sensitivity for recurrence and metastasis, and is therefore an ideal method for monitoring the status of the disease.

## References

[1] Desch CE, Benson AB, Somerfield MR, American Society of Clinical Oncology (2005). Colorectal cancer surveillance: 2005 update of an American Society of Clinical Oncology practice guideline. J Clin Oncol.

[2] Elias D, Sideris L, Pocard M (2004). Results of R0 resection for colorectal liver metastases associated with extrahepatic disease. Ann Surg Oncol.

[3] Lin JK, Lin CC, Yang SH (2011). Early postoperative CEA level is a better prognostic indicator than is preoperative CEA level in predicting prognosis of patients with curable colorectal cancer. Int J Colorectal Dis.

[4] Dirisamer A, Halpern BS, Flöry D (2010). Performance of integrated FDG-PET/contrast-enhanced CT in the staging and restaging of colorectal cancer: comparison with PET and enhanced CT. Eur J Radiol.

[5] Zhang C, Chen Y, Xue H (2009). Diagnostic value of FDG-PET in recurrent colorectal carcinoma: a meta-analysis. Int J Cancer.

[6] Zhang Y, Feng B, Zhang GL (2014). Value of ^18^F-FDG PET-CT in surveillance of postoperative colorectal cancer patients with various carcinoembryonic antigen concentrations. World J Gastroenterol.

[7] Panagiotidis E, Datseris IE, Rondogianni P (2014). Does CEA and CA 19–9 combined increase the likelihood of 18F-FDG in detecting recurrence in colorectal patients with negative CeCT?. Nucl Med Commun.

[8] Makis W, Kurzencwyg D, Hickeson M (2013). 18F-FDG PET/CT superior to serum CEA in detection of colorectal cancer and its recurrence. Clin Imaging.

[9] Tan E, Gouvas N, Nicholls RJ (2009). Diagnostic precision of carcinoembryonic antigen in the detecetion of recurrence of colorectal cancer. Surg Oncol.

[10] Ding Q, Cheng X, Yang L (2014). PET/CT evaluation of response to chemotherapy in non-small cell lung cancer: PET response criteria in solid tumors (PERCIST) versus response evaluation criteria in solid tumors (RECIST). J Thorac Dis.

[11] Litière S, de Vries EG, Seymour L, RECIST Committee (2014). The components of progression as explanatory variables for overall survival in the Response Evaluation Criteria in Solid Tumours 1.1 database. Eur J Cancer.

[12] Esteves FP, Schuster DM, Halkar RK (2006). Gastrointestinal tract malignancies and positron emission tomography: an overview. Semin Nucl Med.

[13] Nielsen HJ, Jess P, Aldulaymi BH (2013). Early detection of recurrence after curative resection for colorectal cancer - obstacles when using soluble biomarkers?. Scand J Gastroenterol.

[14] Liu FY, Chen JS, Changchien CR (2005). Utility of 2-fluoro-2-deoxy-D-glucose positron emission tomography in managing patients of colorectal cancer with unexplained carcinoembryomic antigen elevation at different levels. Dis Colon Rectum.

[15] Moertel CG, Fleming TR, Macdonald JS (1993). An evaluation of the carcinoembryonic antigen (CEA) test for monitoring patients with resected colon cancer. JAMA.

[16] Zerhouni EA, Rutter C, Hamilton SR (1996). CT and MR imaging in the staging of colorectal carcinoma: report of the Radiology Diagnostic Oncology Group II. Radiology.

[17] Ozkan E, Soydal C, Araz M (2012). The role of 18F-FDG PET/CT in detecting colorectal cancer recurrence in patients with elevated CEA levels. Nucl Med Commun.

[18] Chiewvit S, Jiranantanakorn T, Apisarnthanarak P (2013). Detection of recurrent colorectal cancer by 18F-FDG PET/CT comparison with contrast enhanced CT scan. J Med Assoc Thai.

[19] Peng NJ, Hu C, King TM (2013). Detection of resectable recurrences in colorectal cancer patients with 2-[18F]fluoro-2-deoxy-D-glucose-positron emission tomography/computed tomography. Cancer Biother Radiopharm.

[20] Lu YY, Chen JH, Chien CR (2013). Use of FDG-PET or PET/CT to detect recurrent colorectal cancer in patients with elevated CEA: a systematic review and meta-analysis. Int J Colorectal Dis.

[21] Lee JE, Kim SW, Kim JS (2012). Prognostic value of 18-fluorodeoxyglucose positron emission tomography-computed tomography in resectable colorectal cancer. World J Gastroenterol.

[22] Choi EK, Yoo IeR, Park HL (2012). Value of Surveillance (18)F-FDG PET/CT in Colorectal Cancer: Comparison with Conventional Imaging Studies. Nucl Med Mol Imaging.

[23] Sanli Y, Kuyumcu S, Ozkan ZG (2012). The utility of FDG-PET/CT as an effective tool for detecting recurrent colorectal cancer regardless of serum CEA levels. Ann Nucl Med.

[24] Maas M, Rutten IJ, Nelemans PJ (2011). What is the most accurate whole-body imaging modality for assessment of local and distant recurrent disease in colorectal cancer? A meta-analysis: imaging for recurrent colorectal cancer. Eur J Nucl Med Mol Imaging.

[25] Mittal BR, Senthil R, Kashyap R (2011). 18F-FDG PET-CT in evaluation of postoperative colorectal cancer patients with rising CEA level. Nucl Med Commun.

[26] Schmidt GP, Baur-Melnyk A, Haug A (2009). Whole-body MRI at 1.5 T and 3 T compared with FDG-PET-CT for the detection of tumor recurrence in patients with colorectal cancer. Eur Radiol.

[27] Simó M, Lomeña F, Setoain J (2002). FDG-PET improves the management of patients with suspected recurrence of colorectal cancer. Nucl Med Commun.

